# Fatal Idiopathic Hyperammonemia after Induction Chemotherapy for Acute Myeloid Leukemia

**DOI:** 10.1155/2020/3136074

**Published:** 2020-02-08

**Authors:** Christophe Angelo, Marie-Françoise Vincent, Mina Komuta, Philippe Hantson, Nicole Straetmans, Edwige Boulet

**Affiliations:** ^1^Department of Intensive Care, Cliniques St-Luc, Université Catholique de Louvain, 1200 Brussels, Belgium; ^2^Department of Clinical Chemistry, Cliniques St-Luc, Université Catholique de Louvain, 1200 Brussels, Belgium; ^3^Department of Pathology, Cliniques St-Luc, Université Catholique de Louvain, 1200 Brussels, Belgium; ^4^Louvain Centre of Toxicology and Applied Pharmacology, Université Catholique de Louvain, 1200 Brussels, Belgium; ^5^Department of Hematology, Cliniques St-Luc, Université Catholique de Louvain, 1200 Brussels, Belgium

## Abstract

Idiopathic hyperammonemia is a rare but potentially fatal complication occurring in patients with acute leukemia or bone marrow transplantation. The role of some specific anticancer drugs may be discussed, but the etiology of hyperammonemia is often multifactorial. We report the case of a 40-year-old woman who developed fatal idiopathic hyperammonemia two weeks after induction chemotherapy with idarubicin-aracytine for acute myeloid leukemia. Despite intensive care management and extrarenal epuration, the patient was declared brain dead two days after hyperammonemia onset.

## 1. Introduction

Idiopathic hyperammonemia (IHA) has been described for the first time in the 1980's after induction chemotherapy for acute leukemia [1]. It has also been occasionally reported in patients receiving bone marrow transplantation or suffering from multiple myeloma [2–4]. This complication occurs often with some delay after the start of chemotherapy, in the absence of underlying liver disease. Among the medications possibly associated with IHA, cytarabine has been frequently cited [1, 5]. As in the here reported observation, the role of other precipitating factors may also be debated. The possible involvement of parenteral nutrition or intestinal microbial proliferation is discussed.

## 2. Case Presentation

**Figure 1:**
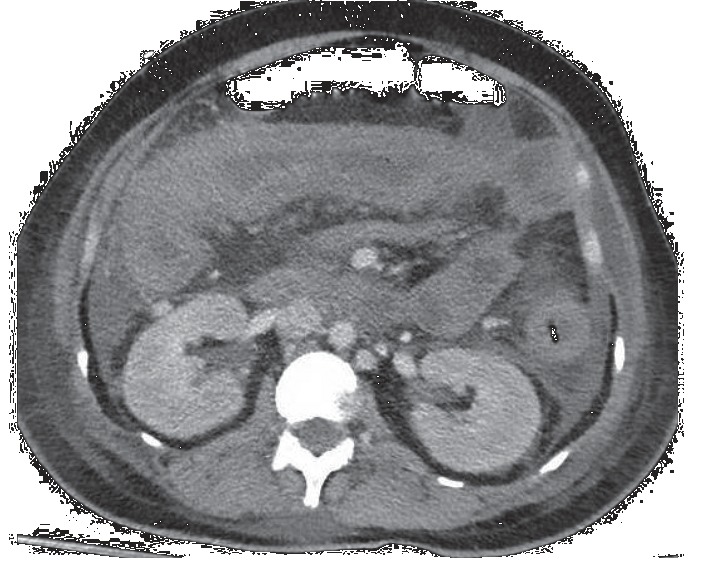
Contrast-enhanced abdomen computed tomography with oedematous thickening of the caecal and colonic mucosa.

A 40-year-old woman without previous medical history was admitted to the Haematology Department for progressive fatigue and grade 2-3 dyspnoea which appeared one month before. The physical examination found a patient in a good general condition, but with extreme pallor. Laboratory investigations revealed hemoglobin 5.8 g/dl, white blood cell count 6040/mm³ with 36% blasts, and platelet count 58,000/mm³. Liver function tests were normal. The diagnosis of acute myeloid leukemia (AML) with inv(16)(p13.1q22), CBF-MYH11 (FISH analysis) was obtained. The induction chemotherapy included idarubicin 12 mg/m^2^ (days 1–3) and aracytine 200 mg/m^2^ (days 1–7). The diagnosis of acute myeloid leukemia (AML) with inv(16)(p13.1q22), CBF-MYH11 (FISH analysis) was obtained. On day 6, after the initiation of chemotherapy, the patient developed neutropenic fever and empiric treatment with piperacillin/tazobactam was started. She developed cytarabine-induced skin lesions and diffuse pancolitis with ascites. This was confirmed by the findings of the contrast-enhanced abdomen computed tomography (CT) that showed a major oedematous involvement of the caecal mucosa (22 mm) and of the right colonic mucosa; there was also a diffuse distension (3 cm diameter) of the small intestine with a thickening of the last loops (Figure 1). Due to this abdominal complication, parenteral nutrition (Aminomix Novum 3, Fresenius, 1500 ml/day, corresponding to 12 gN) was begun on day 10. The patient was transferred to the intensive care unit (ICU) on day 13 for tachycardia, hypotension, and respiratory distress. The abdomen was distended with absence of bowel sounds. The patient was conscious and oriented, and neurological testing was normal. Chest-X-ray examination did not reveal pneumonia. Arterial blood gas analysis showed pH 7.30, pCO_2_ 32 mmHg, and bicarbonate 20 mmol/l. Serological testing for HBV and HCV antibodies was negative; CMV IgG were >500 U/l. Antimicrobial therapy was switched for ceftazidime and vancomycin after the identification of *Enterococcus faecium* in bronchoalveolar lavage and blood culture. Hemodynamics remained stable, without need of vasopressors, and repeated blood culture was sterile. Stool culture revealed Gram-positive flora, but no *Clostridium difficile* nor other pathogens, with the limitation that stool culture was not repeated. On day 16, there was an unexpected impairment of consciousness leading to intubation for coma progression. The plasma ammonia level was 688 *μ*mol/l, with the other relevant laboratory investigations displayed in Table 1. Abdomen ultrasound examination excluded the presence of a portal-systemic shunt. The brain computed tomography did not demonstrate cerebral edema. The electroencephalogram confirmed severe slowing of electrical activity (delta waves, 4 Hz), but without triphasic waves. Analysis of serum amino acids revealed high levels of glutamine, low levels of citrulline, and normal levels of ornithine. There was an extremely high glutamine concentration (7976 *μ*mol/l) into the cerebrospinal fluid (CSF). Parenteral nutrition was interrupted, without any possibility of enteral feeding. Continuous venovenous hemofiltration (CVVH) was started and completed by intermittent hemodialysis, without significant efficacy on ammonia clearance. The patient received also intravenous carnitine supplementation (100 mg/kg/d), lactulose intrarectally, and two doses of rifaximin 550 mg via the nasogastric tube. Coma persisted, with a Glasgow Coma Scale score at 3/15 and delta-wave coma but no seizure activity at repeat EEG. Other causes of encephalopathy were excluded (uremic or sepsis-associated encephalopathy, infectious encephalitis, etc.). According to clinical and electrophysiological criteria, the patient was declared brain dead on day 18 after the start of chemotherapy. At postmortem examination, the liver architecture appeared relatively well preserved with about 10–20% of the hepatocytes presenting ballooning and nuclear loss in the pericentrolobular area. There was no evidence of intestinal necrosis.

## 3. Discussion

This patient developed fatal hyperammonemic encephalopathy less than 3 weeks after the start of induction chemotherapy based on the combination cytarabine-idarubicin to treat a recently diagnosed AML. This delay is consistent with a previous case who developed a similar clinical course on day 21 after having received high-dose cytarabine and mitoxantrone for AML and with other literature cases, even if a shorter delay (a few hours) was occasionally observed [6, 7]. The true incidence of IHA in patients receiving chemotherapy for acute leukemia is not known, as ammonia levels are usually not measured in asymptomatic patients, but reached 2.4% in the largest literature series [8]. Most of these cases occurred in the absence of severe hepatic dysfunction and other causes of hyperammonemia could also be ruled out. Therefore, this hyperammonemia is often classified as idiopathic [1, 2]. We can however assume that most of the systemic ammonia pool is originally generated in the gastrointestinal tract and this was likely the case in the present observation. The clinical presentation is usually characterized by rapidly progressive signs of central nervous system dysfunction including coma and seizures [9]. The prognosis is usually extremely poor, with a mortality rate around 80% [1, 2]. At postmortem examination, brain swelling and herniation are commonly observed. Our observation confirmed the presence of high glutamine levels in the CSF; as a metabolite of ammonia, the intracellular accumulation of glutamine may be responsible for an osmotic effect with astrocyte swelling [1]. The liver is usually free from preexisting disease. Ultrastructural liver changes differ from those observed in Reyes syndrome and usually reveal congestion, fatty infiltration, and cholestasis.

The origin of hyperammonemia is likely multifactorial. Cytarabine itself contributes very slightly to ammonia formation through a metabolic process of deamination [4]. Among other chemotherapy agents, 5-fluorouracil (5-FU) has also been associated in some cases with hyperammonemia [6]. However, ammonia is a product of 5-FU metabolism, and the clinical course of hyperammonemia is usually transient and benign. L-asparaginase is also often related to IHA with a mechanism that is also not fully understood [10]. There is insufficient evidence to prove that a congenital metabolic disorder is responsible for hyperammonemia. For the patients explored by plasma amino acid determinations, no specific profile could be identified. In our case, a urea cycle defect was unlikely based on slightly low plasma levels of citrulline, but normal plasma levels of ornithine and arginine, absence of argininosuccinate, and normal urinary levels of orotic acid [11]. Our patient had also no evidence of a preexisting congenital liver disease (Wilson's disease, for example). The role of some additional precipitating factors may also be discussed. Among them, sepsis, dehydration, and nitrogen load by parenteral nutrition are commonly found and were present in our patient [1, 9, 12]. The ammonia production could also have been increased by the ileus-related microbial proliferation [13]. Reports of hyperammonemia due to urease-producing bacteria such as *Klebsiella spp*., *Proteus spp.*, *Corynebacterium spp*., and *Staphylococcus spp.* are commonly found, while *Enterococcus faecium* is not frequently cited [14].

Several strategies have been proposed to control hyperammonemia (Table 2) [15]. Some pharmacological agents (sodium benzoate and sodium phenylacetate) have been proposed to promote the elimination of ammonia, with variable outcomes [16]. In addition to ammonia-trapping agents, hemodialysis should be initiated early to promote ammonia elimination [17]. It has been suggested to continue hemodialysis until the blood ammonia concentration has dropped below 200 *μ*mol/l for a period of at least 24 hours. Continuous venovenous hemofiltration (CVVH) is a possible alternative to hemodialysis. Even if it appears less effective than hemodialysis, CVVH is a continuous method that could be maintain for a longer period and could clear newly produced nitrogen [18]. However, these techniques may be limited by ammonia overproduction. The molecular adsorbent recirculating system (MARS) is an epuration technique based upon albumin dialysis. It could promote the elimination of bilirubin, bile acid, ammonia, and cytokines. It has been used with some efficacy in a case of L-asparaginase-related hyperammonemia [19]. Adequate nutritional support is essential to avoid protein catabolism or excessive protein intake by parenteral nutrition. Any medication impairing intestinal motility should be avoided. Finally, the long-term oral administration of a strain (SF68) of *Enterococcus faecium* was able to reduce the production of ammonia and the severity of encephalopathy in a single study of patients with cirrhosis and a normal dietary nitrogen intake [20]. Other strains (*Lactobacillus*, *Bifidobacterium*, etc.) have been tested using the same probiotic approach [15].

## Figures and Tables

**Table 1:** Laboratory data before and after hyperammonemic coma.

	Normal range	Day 1 (start of chemotherapy)	Day 7 (end of chemotherapy)	Day 13 (ICU admission)	Day 16 (coma onset)	Day 17	Day 18 (death)
Maximal daily temperature (°C)		36.6	38.4	38.8	37.3	37.2	35.8
CRP (mg/l)	<5	2.6	236.4	463.8	313.6	292.1	340.5
Ammonia (*μ*mol/l)	<64	n.d.	n.d.	n.d.	688	1989	992
Total bilirubin (mg/dl)	<1.2	0.5	0.8	5.8	6.5	5.7	6.4
Alkaline phosphatase (IU/l)	35–105	40	28	21	35	37	42
Gamma-glutamyl transpeptidase (IU/l)	<40	16	46	28	32	36	41
Aspartate aminotransferase (IU/l)	15–35	12	12	45	28	37	42
Alanine aminotransferase (IU/l)	7–35	12	9	31	22	21	23
International normalized ratio	0.80–1.20	1.15	1.53	1.64	1.39	1.42	1.46
Arterial lactate (mmol/l)	0.5–2.2	n.d.	n.d.	2.8	2.1	2.9	2.7
Triglycerides (mg/dl)	<150	n.d.	n.d.	n.d.	n.d.	513	390

n.d., not done.

**Table 2:** Common therapeutic options for hyperammonemia.

Intervention	Mechanism
Lactulose	Acceleration of intestinal transit time
Intestinal antibiotics (digestive decontamination)	Reduction of intestinal microbial proliferation
Administration of probiotics	Changes in intestinal microbiota
Sodium benzoate/phenylacetate	Conjugation with glycine to form hippuric acid and promote urea excretion
Carnitine	Regulation of fatty acids metabolism
Reduction in protein intake, avoidance of catabolism	Decreased activity of urea cycle and ammonia production
Epuration techniques (HD, CVVH, MARS)	Increased ammonia elimination
